# Research in Nonlinearity of Surface Acoustic Wave Devices

**DOI:** 10.3390/mi12121454

**Published:** 2021-11-26

**Authors:** Yahui Tian, Litian Wang, Yuanyuan Wang, Yang Li, Haoxiang Wu, Lirong Qian, Honglang Li, Jinghui Wu, Ji Wang

**Affiliations:** 1 Institute of Acoustics, Chinese Academy of Sciences, Beijing 100190, China; tianyahui@mail.ioa.ac.cn; 2 School of Integrated Circuit Science and Engineering, Tianjin University of Technology, Tianjin 300384, China; mr_wangyuanyuan@163.com; 3 National Center for Nanoscience and Technology, Beijing 100190, China; liy2020@nanoctr.cn; 4 Faculty of Mechanical Engineering & Mechanics, Ningbo University, Ningbo 315832, China; 1911081013@nbu.edu.cn (H.W.); emmawjh@foxmail.com (J.W.); wangji@nbu.edu.cn (J.W.)

**Keywords:** surface acoustic wave, nonlinearity, RF system, filters

## Abstract

Surface acoustic wave (SAW) devices are one of the indispensable components in the radio frequency (RF) front-end of mobile phones. With the development of mobile communication technology, the requirements for linear specification of devices are more and more strict. Nonlinear distortions of SAW devices have a serious influence on the application of mobile RF modules. To satisfy the strict requirement of linearity of communication system, it is necessary to understand the generation mechanism of nonlinearity and study the accurate modeling, appropriate measurement methods, and nonlinear response elimination technology. In this paper, we summarize the research progress on the nonlinearity of SAW devices in recent years from four aspects: the generation mechanism, simulation methods, measurement system, and suppression technology. The nonlinear harmonics with the nonlinear Mason equivalent circuit model are simulated. Furthermore, harmonics and intermodulation signals of SAW filters are tested by the authors. Thanks to these research studies, it is of great significance to the development of future RF front-end modules with high linear SAW devices.

## 1. Importance of Study on Nonlinear Effects in Surface Acoustic Wave Devices

Surface acoustic wave (SAW) devices use the piezoelectric and inverse piezoelectric properties of piezoelectric materials, which excite and receive SAW through an Interdigital transducer (IDT). Their most extensive applications include SAW sensors [1,2] in the Internet of Things and SAW filters [3,4] in mobile communication. As an indispensable component for mobile radio frequency (RF) front-end, SAW filters, and duplexers have a huge market.

Their common design characteristics include low passband insertion losses, high stopband rejection levels, and high signal isolation between transmitting (Tx) and receiving (Rx) signals. Traditionally, SAW filters are regarded as passive devices with linear signal processing performance ignoring nonlinear effects. However, with the development of third, fourth, and fifth-generation mobile communication networks, the complexity of RF circuits in mobile phones has increased dramatically, requiring mobile phones to handle more and more frequency bands. This is for increasing users with complex modulation/demodulation schemes and higher data rates (such as carrier aggregation technology, etc.). As a result, this forces developers of RF front-end devices, including SAW duplexers, to shrink components sizes.

In particular, the nonlinear requirements of Frequency Division Duplexing (FDD) systems are extremely important because the Tx and Rx signals in the duplexer operate at different frequencies simultaneously within a small area [3]. In FDD systems, the output signal of the transceiver is amplified by a power amplifier (PA) and then filtered by the Tx filter of the duplexer. The filtered signal is connected to the antenna (ANT) via a switch and then propagated into the air. Meanwhile, ANT picks up a signal from the air in the Rx band. The Rx signal is filtered by the Rx filter and then amplified by the low noise amplifier. Finally, the Rx signal is demodulated by the transceiver. The coexistence of Tx and Rx signals can cause many problems. For example, the Tx and Rx isolation of the duplexer is limited, the Tx signal leaks into the receiver and is probably the strongest interference for the receiver in the phone. The deterioration becomes severe when the power amplifier is operating in high power mode.

The nonlinearity of the SAW filters can cause much influence on the RF front-end, which can be classified as follows [4]:

(1) Signal quality degradation: The main feature of signal quality is the specification of Error Vector Magnitude. This norm is used to measure the distance between the constellation points of the actual signal and its ideal position (the ideal position refers to the position of the constellation from which the signal is sent by the ideal transmitter or received by the ideal receiver).
(2) New Spectrum Generation: Harmonics, second-order intermodulation distortion (IMD2), third-order intermodulation distortion (IMD3), and adjacent channel leakage ratio are the most common criteria used to evaluate the severity of spectral increase.
(3) Desensitization: Desensitization is defined as the loss of receiver sensitivity due to the presence of Tx signals. The causes of Rx desensitization may be:
  (A) Rx noise generated by power amplifiers;
  (B) Tx leaks into the receiver due to the limited isolation of Tx and Rx, resulting in DC and RF frequency components falling into the receiving band of the direct-conversion receiver;
  (C) IMD2 and IMD3 fall in the Rx frequency band in the duplexer, thus reducing the SNR of the receiver. These IMDs are caused by intermodulation between Tx signals and external jamming signals (Jammers).

In general, the nonlinear distortion of SAW filters has a great impact on the application of mobile devices. Three factors contribute to the emergence and non-negligibility of nonlinear effects of SAW devices: (1) Realization of carrier aggregation between different bands; (2) Miniaturization leads to an increase in energy density; (3) Increase of mobile phone drive power. 

To meet the strict linearity requirements of SAW devices, it is necessary to understand the generation mechanism of nonlinearity and study accurate analysis modeling methods, appropriate measurement, and elimination techniques. In recent years, many researchers have contributed to these studies. Previous works are mainly on the generation mechanism of nonlinearity, simulation methods, measurement systems, and suppression technology. In the following, we have made the conclusion for these studies. 

## 2. Generation Mechanism of Nonlinearity

To study the accurate analysis modeling method and realize improvement of linearity in SAW devices without deteriorating the other performances, we should understand the generation points and extensive mechanisms of nonlinear signals as a first step.

As early as 1989, Hosaka etc. from Hitachi Ltd. (Tokyo, Japan) first studied the intermodulation phenomenon in SAW filters for cellular radio systems, indicating the intermodulation being closely related to the applied power and temperature [5].

**Figure 1:**
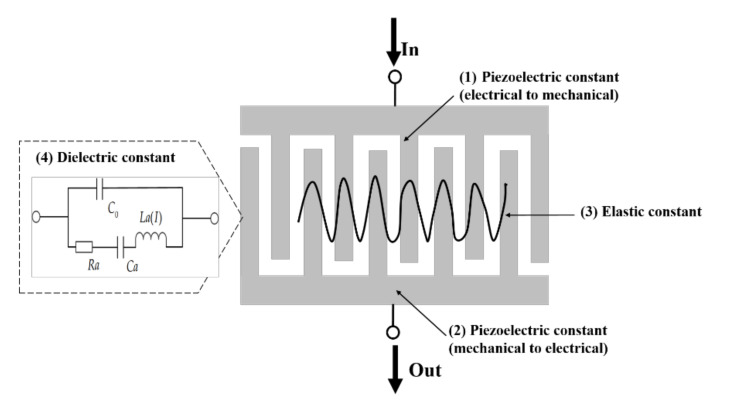
Possible causes of nonlinearity in SAW devices.

The source of the nonlinearity may refer to the mechanisms from the piezoelectric constitutive equations [6]. As they satisfy the piezoelectric constitutive equations:

(1) $$T=cS-e_{1}.E\;D=e_{2}S+\varepsilon E$$

where *T* is the stress, *D* is the electric displacement, *S* is the strain, and *E* is the electric field. There are four coefficients in Equation (1) and thus there will be four possible causes of the nonlinearity in SAW resonators as shown in Figure 1. That is: (1) nonlinearity of piezoelectric constant (electrical to mechanical), (2) nonlinearity of piezoelectric constant (mechanical to electrical), (3) nonlinearity of elastic constant, and (4) nonlinearity of dielectric constant.

In this equation, all these four constants include second-, third- and fourth-order material constants. Such as the *c* include *c_ijkl_*, *c_ijklmn_*, *c_ijklmnpq_*, the terms *c_ijkl_*, *c_ijklmn_*, *c_ijklmnpq_* are the second-, third-, and fourth-order elastic constants. Similarly, the piezoelectric constants and dielectric constant also include second-, third- and fourth-order constants. For the second harmonic, it is derived by the second- and third-order constants of both these four constants. For the third harmonic, it is derived by the second-, third- and fourth-order constants of both these four constants.

Apart from the possible causes, generation points of nonlinear signals [7,8,9,10,11,12,13,14], whether in the substrate or in electrodes, are also important. Previous works show that the second-order nonlinearity in SAW devices on 42°XY-LiTaO_3_ (42-LT) substrates is mainly generated by the crystalline asymmetry on the surface of a piezoelectric substrate, and the nonlinear dielectric constant and the electrostrictive constant contribute to the generation of second-order nonlinear signals. Furthermore, the following two mechanisms mainly contribute to their generation: (a) self-mixing of the electrostatic field and (b) mixing of the electrostatic field with the strain field associated with laterally propagating modes. Both of them occur at the gaps between the electrode tip and the dummy electrode [7,8]. However, the third-order is closely related to the acoustic strain in the aluminum electrode, including the higher the crystallinity, the more obvious the nonlinear. When the electrode is laminated, the nonlinearity is suppressed. Furthermore, the width of the electrode also has a great influence on the nonlinear effect. Usually the wider the electrode width, the more obvious the nonlinear effect [9,10,11,12,13,14].

Different from common SAW devices, the nonlinearity mechanism of temperature compensated SAW (TCSAW) devices with silicon dioxide on the Lithium Niobate are different from those published for leaky SAW resonators on Lithium Tantalate. For the second harmonic, strong peaks looking like multiple resonant modes are found. The frequency difference between the successive peaks increases when the substrate thickness decreases. Results also show that the level of the signal depends on the roughness of the back side of the substrate [15]. Due to these findings, it was assumed that this signal is due to the nonlinear generation of a bulk mode. This assumption was confirmed experimentally by scanning the back side with an interferometer by Solal et al. [15].

## 3. Simulation Method of Nonlinearity in SAW Devices

Traditionally, the passive SAW devices and bulk acoustic wave (BAW) are regarded as linear devices. Thereby, the design methodology of SAW devices is usually based on linear models including P-matrix [16] or coupling of modes (COM) model [17,18], and so on. However, owing to the nonlinear specifications of 3GPP, the design method of SAW duplexers must include nonlinear analysis and simulation. Thus, to achieve the analysis of nonlinear characteristics, serval phenomenological models have also been developed, such as nonlinear Butterworth Van Dyke (BVD) model [3], nonlinear Mason equivalent model [4,19], nonlinear P-matrix model [20,21], nonlinear COM model [22,23,24,25], rigorous nonlinear COM and P matrix model [26,27,28,29] and other nonlinear phenomenological models [30,31,32,33,34,35]. Furthermore, a finite element algorithm model was also developed for the nonlinear analysis [36,37,38].

Although both SAW and BAW filters are passive devices, the nonlinear characteristics of SAW devices differ from those of BAW devices due to different waveforms, excitation, propagation, and geometry on the piezoelectric substrate. Since there are two electrodes per acoustic wavelength, SAW resonators have better inherent second-order nonlinearity than BAW devices. However, they have the worst terms of third-order nonlinearity. Therefore, most previous studies of SAW nonlinearity focused on the third-order nonlinearity effect. 

### 3.1. Phenomenological Models

The origin of the nonlinear characteristics is commonly believed that the nonlinearity of the SAW resonator is mainly owing to the nonlinear effect of the piezoelectric material of the resonator. Nevertheless, the nonlinear constant of piezoelectric materials is hardly measured. Unfortunately, as far as the scope of our knowledge, only the Lithium Niobate and quartz have been reported in the references [39,40,41] for their measured value of nonlinear material constants. Thus, it is difficult to simulate the nonlinear acoustic field from the piezoelectric constitutive equations. Furthermore, the estimation of the main nonlinear mechanism is not feasible based on the physical constants of the material for researchers. 

Thus, like the traditional linear analysis for SAW devices, phenomenological models are more useful for common devices. Phenomenological models do not use nonlinear material constants. They just need the equivalent nonlinear constant from the experimental measurement. They are efficient for the design of SAW devices.

#### 3.1.1. Nonlinear BVD Model

**Figure 2:**
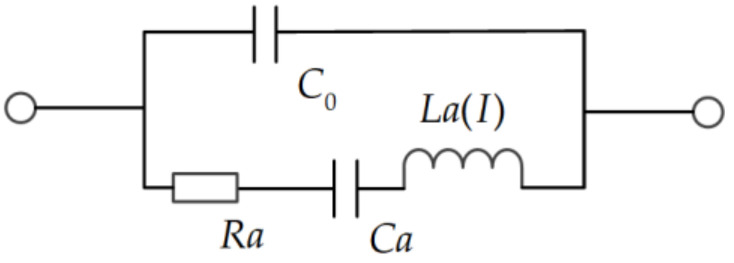
The schematic diagram of nonlinear BVD model.

In 2010, a nonlinear BVD model was developed and demonstrated by Li Chen et al. [3]. The nonlinear BVD model is a relatively simple method to realize the nonlinear effect analysis. It is developed by the linear BVD model with adding the nonlinear term into the standard model as shown in Figure 2.

To analyze the third-order nonlinear effects in the SAW resonators and duplexers, methods are demonstrated as below:

Firstly, starting from the linear BVD model, the nonlinear simulation of the SAW resonator is completed by assuming the quadratic dependence of the dynamic inductance (*La*) on the current root mean square *I*. Thereby, the dynamics inductance (*La*) changes with the current can be expressed as:

(2) $$L_{a}\left(I\right)\;=L_{a}\times \left(1+L_{a3}\cdot I^{2}\right)$$

Herein, *L_a3_* is the third-order coefficient of the inductance. 

The nonlinear BVD model can be directly simulated by using the circuit simulation software. In the simulation software, a harmonic balance simulator is adopted to predict the nonlinear response of the BVD equivalent circuit. The simulation techniques mainly include the following steps: firstly, extract all parameters directly except *L_a3_* of the BVD model from the measurement results of a small signal. Secondly, deriving *L_a3_* by fitting the harmonic simulation results with measured dynamic inductance. Usually, *L_a3_* depends on the size of the resonator and the metallization rate of IDT. At last, the model can be verified by accurately simulating the third harmonic and IMD3 of the resonator and duplexer. 

Previous results show the simulated results of duplexer nonlinear distortion based on the BVD model agree well with the measurement results. Furthermore, it is easy to integrate into the simulation tool.

However, parameters in the nonlinear BVD model need to be extracted from each resonator in the duplexer. Therefore, a large number of experiments should be conducted. Additionally, the nonlinear BVD model is not suitable for analyzing devices with coupled-resonator filters, which is commonly used in the SAW duplexers.

#### 3.1.2. Nonlinear Mason Equivalent Circuit Model

Afterwards, the nonlinear Mason equivalent circuit model was proposed [4,19] as it is more precise than the nonlinear BVD model. 

**Figure 3:**
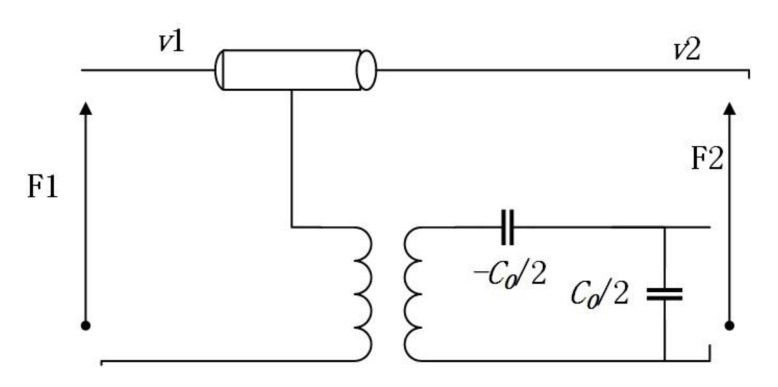
The Schematic diagram of nonlinear Mason equivalent circuit model.

In the Mason equivalent circuit model, as can be seen from Figure 3, the electrode part of the IDT can be represented by an equivalent electromechanical circuit with lumped elements. The lumped elements of the Mason equivalent circuit model can also be replaced by transmission lines which is also called the Redwood version of the Mason model. The form of distributed transmission lines is adopted in the Mason circuit model to facilitate the analysis and demonstration of the IDT structure on the piezoelectric substrate. Owing to the Mason model containing controllable piezoelectric constitutive equations, Newton equations of motion, and Gauss’ law, the Mason model is an accurate physical representation of the propagation of acoustic waves. Additionally, it can be found from the Mason model that the propagation of the higher-order waves can be modeled by extending the linear Mason model with nonlinear piezoelectric material properties. 

The Redwood version of the linear mason circuit model is depicted in Figure 3, which stands for an electrode part of the IDT. There are three ports that can be seen in Figure 3. Herein, Port 1 and Port 2 are acoustic ports. (*F*_1_, *v*_1_) and (*F*_2_, *v*_2_) separately represent the mechanical force and speed of the left and right edges. Port 3 is an electrical port with excitation voltage *U*_3_ and current *I*_3_. Moreover, the transformer turns ratio *ϕ* denotes piezoelectric coupling, *C*_0_ stands for the static capacitance and 3-ports network contains the electromechanical relationship of IDT.

Generally, the Mason model is a common model which operated with parameters set. And the parameters only depend on the material type of the substrate but not the specific geometry of the transducers. 

Major characteristics of the nonlinear Mason model are demonstrated as follows: 

Firstly, the nonlinear Mason model is the first physical model derived from the physical principles of acoustic wave propagation. The nonlinear Mason model allows the simulation of nonlinear effects of SAW resonators and duplexers in a flexible and easy to configure manner based on the equivalent circuit method. Therefore, the nonlinear Mason model can be directly applied to commercial circuit simulators without any mathematical conversion. Additionally, the high order nonlinear parasitic responses based on S-parameters and voltage-current can be simulated simultaneously by employing the same equivalent circuit. As far as we know, the nonlinear Mason model is the unique equivalent circuit that can be directly implemented in commercial circuit simulators. It can be as accurate as other nonlinear physical models. Similarly, the nonlinear Mason model has a compatible interface that can be co-simulated with other front-end components such as PA and RF switches by utilizing the harmonic balanced simulator. Therefore, this is a significant advantage of the nonlinear Mason equivalent circuit model. Besides, the S-parameter of the duplexer also provides convenience for checking the insertion loss of the duplexer and the impedance mismatch between components.

Secondly, the nonlinear Mason equivalent circuit model is recognized as the easiest method to predict nonlinear effects. The establishment of the nonlinear Mason model with the help of circuit simulation software, such as ADS, is convenient. This is owing to the unit section of the model being an electrode, which only contains several elements and a constant nonlinear coefficient. The simulation of the full duplexer can be accomplished within a few minutes. Compared with the other nonlinear physical models, time cost required is reduced by using the nonlinear Mason circuit model. 

However, the Mason equivalent circuit model is derived from the piezoelectric constitutive equation. Thus, the nonlinear Mason equivalent circuit model is a one-dimensional equivalent circuit, and several assumptions are used in terms of acoustic wave propagation.

**Figure 4:**
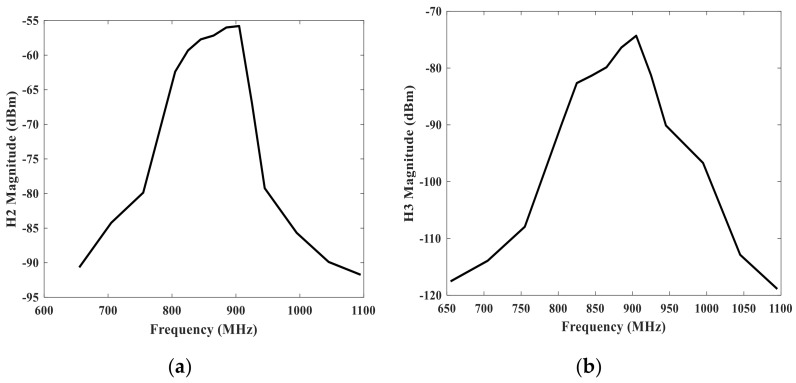
Results of harmonics of SAW resonators with nonlinear Mason equivalent circuit model. (**a**) the second harmonic response H2. (**b**) the third harmonic response H3.

The authors in this paper also developed the nonlinear Mason equivalent circuit model and simulated the harmonics in the SAW resonators with a center frequency of 880 MHz as shown in Figure 4. Firstly, we established the circuit model based on the Mason model. Based on finite element simulation, we obtained the electrical parameters under the Mason model from the structural parameters and material parameters of a single IDT. Then we cascade 40 pairs and implement a single IDT resonator model. Afterwards, we introduced the nonlinear constants into the model. Finally, the harmonic balance simulator is used in the model to simulate the second harmonic (H2) response and third harmonic (H3) response. The results shown in Figure 4 agree with the results shown in [4].

#### 3.1.3. Nonlinear P Matrix Model

As demonstrated and reported by Solal and Chen Li et al. [20,21], the nonlinear P matrix is recognized as a nonlinear wave model. This model constructs a nonlinear matrix by assuming that the propagation of SAW on the substrate can be expressed by the following equation:

(3) $$\frac{\partial^{2}s}{\partial t^{2}}=v^{2}\frac{\partial^{2}}{\partial x^{2}}\left(s+\beta s^{2}+\gamma s^{3}\right)$$

With the assumption that SAW is represented by a scalar *s*, *s* denotes the mechanical strain, *v* stands for the wave velocity, *β* and *γ* are respectively the second and third-order nonlinear coefficients which are derived from the nonlinear part of the elastic coefficient. Under this circumstance, the third harmonic term is mainly obtained by mixing the second harmonic and fundamental frequency. The nonlinear part of the strain is then added to the regular P matrix.

To solve the nonlinear P matrix, the perturbation method is implemented. Firstly, the standard linear P matrix model is employed to analyze the acoustic on the fundamental frequency of the device. Thus, the admittance of the device and the amplitude of the propagating along the device at these frequencies can be predicted. Secondly, the directly mixed products by second- and third-order are obtained. At this stage, to represent the mixed product generated along the propagation direction, the source term is added to the P matrix, where the source term depends on the wave amplitude at the fundamental frequency. Furthermore, it also includes the normal propagation reflection and conversion at the frequency of the mixed product. 

The realization of the nonlinear P matrix model is accomplished by converting the nonlinear P matrix into a polynomial relationship between harmonic current and harmonic voltage. Thereby, such a matrix relationship can be fitted to the format of the frequency domain devices. Finally, the harmonic balance simulator in the commercial circuit software can recognize the format and simulation of resonator and duplexer can be achieved. Previous work shows the results of the simulation by nonlinear P matrix agree well with the experimental results. 

Unlike the nonlinear Mason equivalent circuit model, nonlinear parameters in the nonlinear P matrix just depend on the material of the substrate. This can reduce many experiments compared with the nonlinear Mason equivalent circuit model.

#### 3.1.4. Nonlinear COM Model

Inoue et al. from Taiyo proposed a nonlinear elastic COM model, which only considered nonlinear elastic constants. They simulated the nonlinear wave proportional to the three tones wave amplitude product and the corresponding current source. This is recognized as a triple-beat product of the SAW duplexer [22,23,24,25].

This nonlinear COM model is based on the third-order nonlinear elasticity of SAW and needs just one nonlinear parameter. This nonlinear parameter represents the third-order nonlinear coefficient for the elastic constant. In their works, results of SAW duplexers for 1.9 GHz personal communications services demonstrated fairly good agreement with the measurements. Results showed an accuracy of less than 1 dB. Results also suggested that the main cause of the triple beat in the SAW duplexer was the nonlinear elasticity of SAW.

Such a nonlinear COM method is quite similar to the nonlinear P matrix model. Although the results presented accuracy, this nonlinear COM model does not account for the reflection of the nonlinearly generated waves. 

#### 3.1.5. Nonlinear Rigorous COM and P Matrix Approach

All the above models have in common to employ a one-dimensional phenomenological model with a nonlinear relation between stress and strain. Compared with these phenomenological models, Mayer et al. further adopted the nonlinear rigorous COM and P matrix approach from first principles [26,27,28,29]. 

Different from these one-dimensional phenomenological models, they started from basic principles, three-dimensional electro-acoustic equations, to derive a set of nonlinear coupled COM equations. These COM equations were translated into an extended P matrix formalism which was used to calculate IMD3 and triple beat of different filters and duplexers in different frequency ranges. The scheme depends on two frequency-independent constants for an effective third-order nonlinearity.

Starting from a nonlinear relationship between the stress and electric displacement field on the one hand and the strains and electric field on the other hand. The resulting approach depends on a single frequency-independent constant. This constant accounts for acoustic dielectric and piezoelectric nonlinearities of propagating waves. 

Usually, a generalized displacement field can be indicated as $u_{m},m=1,\;2,\;3,\;4$. Its first three components are the usual displacement field, while the fourth component represents the electrostatic potential. Likewise, a generalized Piola-Kirchhoff stress tensor $(T_{mN}),m=1,\;2,\;3,\;4,\;N=1,\;2,\;3$ is introduced. The generalized stress tensor can be expanded in powers of generalized displacement gradients $u_{m},N=\partial u_{m}/\partial x_{N}$,

(4) $$T_{mN}=S_{mMnN}u_{n,N}\;+\frac{1}{2}S_{mMnNpP}u_{n,N}u_{p,P}\;+\frac{1}{6}S_{mMnNpPqQ}u_{n,N}u_{p,P}u_{q,Q}$$

In the above equation, summation over repeated indices is implied. The expansion coefficients are material constants. For example, the quantities $S_{mMnNpP}$ are linear combinations of second-order and third-order elastic, piezoelectric, dielectric constants or electrostriction constants. From this equation, they derived the nonlinear COM model. In the COM model, all these nonlinearities are absorbed into one nonlinear parameter. However, nonlinearities of the static capacitances or bulk wave radiation were not included. If we want to account for this, an imaginary part in the nonlinear COM model are needed.

Furthermore, P matrix formalism is extended to account for the nonlinear source terms described above.

(5) $$\left(\begin{array}{c} b_{1}\\ b_{2}\\ i \end{array}\right)=\left[\begin{array}{ccc} P_{11} & P_{12} & E_{1}\\ P_{12}^{T} & P_{22} & E_{2}\\ -2E_{1}^{T} & -2E_{2}^{T} & Y \end{array}\right]\left(\begin{array}{c} a_{1}\\ a_{2}\\ u \end{array}\right)+\left(\begin{array}{c} s_{1}\\ s_{2}\\ s_{u} \end{array}\right)$$

where $a_{1/2}$ and $b_{1/2}$ are power waves entering and leaving, respectively, at the left and right ports. $s_{1/2}$ are the acoustic sources emitting to the left and right ports and $s_{u}$ are the electric current source. In this formalism, the $s_{1/2}$ and $s_{u}$ are the nonlinear part. With this method, the simulation predicted the level and shaped well for the SAW duplexers.

In conclusion, only a single nonlinear parameter is required in this model. As it is started from the basic principles, it is precise. However, this method is difficult to deduce and implement.

#### 3.1.6. Other Nonlinear Models

Besides these, Nakagawa et al. from Murata and Hashimoto et al. [30,31,32,33] proposed a nonlinear simulation method which considered all nonlinear generation mechanisms. By comparing the simulation results with the measured results, it can be found that the contribution of each mechanism to the nonlinear signal changes significantly with the change of the input and output frequency conditions.

In the techniques proposed by Hashimoto et al., the generation mechanism of third-order nonlinearity in SAW devices is discussed in detail. They have developed a simulation method that takes all the three pivotal mechanisms above into consideration to discuss how each mechanism contributes to the generation of nonlinear signals. In this simulation method, the nonlinear stress and electric flux are estimated from the linear field calculated by using traditional mode coupling of modes analysis. Further, the aforementioned effects are respectively embedded into the equivalent circuit model of the target SAW device as the voltage and current source. Finally, the influence of the peripheral circuit has been considered and the nonlinear signal level of the external terminal can be calculated.

The results reveal that when the parameters required for the simulation are correctly determined, the simulation predictions are matching well with the experiment results. On this basis, a series of data measured under various driving and result frequency combinations can be uniquely determined. This is because the contribution of each nonlinear term to the nonlinearity changes significantly according to the driving frequency and the resulting frequency. In summary, the nonlinear mechanism of elastic mechanism is only significant when the driving frequency and the resulting frequency are close to the resonance frequency $f_{r}$, and the mechanism of electromechanical coupling mechanism is significant only when one of the two frequencies is close to $f_{r}$, and the mechanism of dielectric mechanism occurs at all the frequency range. This result reveals that all three mechanisms mentioned above cannot be ignored. And since the most important mechanism is determined for the most complicated driving condition. 

In addition, M Gonzalez Rodriguez et al. [34] in 2020 have applied a new method of analysis for the simulation of harmonics and intermodulation products generation in SAW devices. The method is called the Input-Output Equivalent Source (IOES) and it allows extremely fast and robust simulations of large distributed weak nonlinear circuits. Results showed that it has accuracy and high computing speed.

### 3.2. Precise Simulation Models

Although under the premise that the optimal design of the SAW devices lacks nonlinear material parameters, the phenomenological models have received widespread attention. However, the nonlinear parameters of the phenomenological models require a large amount of experimental measurement results to extract. On the other hand, accurate acoustic field simulation is of far-reaching significance for a deep understanding of the underlying characteristics of the device. Therefore, the development of a precise simulation is necessary.

Mayer et al. [29] have developed the method of finite element method (FEM). FEM was extended to nonlinear analysis. Results show the IMD3 of an infinite periodic array of electrodes on a piezoelectric substrate can be directly simulated in the sagittal plane. This direct approach opens the way for a FEM-based simulation of nonlinearities for finite and generalized structures avoiding the simplifications of phenomenological approaches. Yook Yong and Xiannan Pang et al. [36,37,38] also calculated the harmonics and intermodulation of SAW and BAW devices by employing COMSOL FEM software.

**Figure 5:**
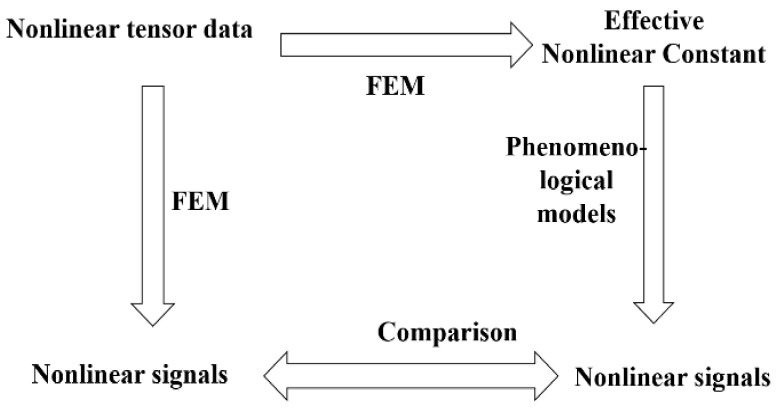
The relation between the FEM model and phenomenological models.

Similar to linear analysis, the accurate acoustic field simulation model will serve the phenomenological models as shown in Figure 5.

All the precise simulation models are based on the basic equations of nonlinear electro-elasticity.

Equations of motion and charge equations of electrostatics is as follows:

(6) $$\left(T_{ij}+T_{jk}U_{i,k}\right),\;j=\rho {\ddot{U}}_{i}\;D_{i},i=0$$

In this piezoelectric constitutive equations, high-order nonlinear terms are included:

(7) $$T_{ij}=C_{ijkl}S_{kl}+\frac{1}{2}C_{ijklmn}S_{kl}S_{mn}\;+\frac{1}{6}C_{ijklmnpq}S_{kl}S_{mn}S_{pq}+\eta_{ijkl}{\dot{S}}_{kl}-e_{kij}E_{k}\;-e_{kijmn}E_{k}S_{mn}-\frac{1}{2}l_{klij}E_{k}E_{l}$$

(8) $$D_{i}=e_{ijk}S_{jk}+\frac{1}{2}e_{ijklm}S_{jk}S_{lm}+\varepsilon_{ik}E_{k}\;+\frac{1}{2}\varepsilon_{ijk}E_{j}E_{k}+l_{ijkl}E_{j}S_{kl}$$

Strain displacement and electric field potential relations are as follows:

(9) $$S_{ij}=\frac{1}{2}(U_{i,j}+U_{j,i}+U_{k,i}U_{k,j})\;E_{i}=-\varphi ,i$$

Mechanical and electrical boundary conditions include:

(10) $$\left(T_{ij}+T_{jk}U_{i,k}\right)n_{j}={\hat{T}}_{i}\;on\;\Gamma_{1}\;U_{i}={\hat{U}}_{i}\;on\;\Gamma_{2}\;\varphi =\hat{\varphi }\;on\;\Gamma_{3}\;n_{i}D_{i}=0\;on\;\Gamma_{4}$$

where $U_{i}$, $T_{ij}$, $S_{kl}$, φ, $E_{i}$ and $D_{i}$ are the mechanical displacement, stress, strain, electric potential, electric field, and electrical displacement fields, respectively. The terms $C_{ijkl}$, $C_{ijklmn}$, $C_{ijklmnpq}$ are the second- third-, and fourth-order elastic constants; $\eta_{ijkl}$ is the viscosity tensor; $e_{kij}$ and $e_{ijklm}$ are the second- and third-order piezoelectric constants; $l_{ijkl}$ are the electrostriction constants; $\varepsilon_{ik}$ and $\varepsilon_{ijk}$ are the second- and third-order dielectric constants; ρ is the mass density, $n_{i}$ is the unit normal vector at the boundaries; ${\hat{T}}_{i}$, ${\hat{U}}_{i}$, and $\hat{\varphi }$ are specified stress, mechanical displacement, and electric potential at certain boundaries, respectively.

Staring from these nonlinear equations, nonlinearly coupled sets of piezoelectric field equations in the frequency domain can be derived. Afterwards, the nonlinear acoustic field and nonlinear effects can be calculated by manual programming or just by using the FEM software. This is easy to be achieved.

### 3.3. Comparison of Different Nonlinear Models

Previous works have conducted many different nonlinear models and demonstrated their accuracy. Different nonlinear models have different characteristics. Phenomenological models are efficient for nonlinear analysis in SAW devices. The simplest nonlinear BVD model is more common in the analysis of BAW devices. As it cannot analyze the device of coupled-resonator filters, it is rarely adopted in the analysis of SAW devices. For nonlinear analysis of SAW devices, the nonlinear Mason equivalent circuit model is a relatively good choice as it is very easy to implement, compared with the nonlinear P matrix and nonlinear COM. However, all these models are one-dimensional phenomenological models. Different from them, the nonlinear rigorous COM and P matrix approach is more precise, which are derived from the basic principle. 

Moreover, precise simulation models are necessary for the nonlinear acoustic analysis of SAW devices. The nonlinear FEM model can simulate the acoustic fields and it is helpful to understand the mechanism of nonlinearity generation. Furthermore, this model can also be the simplest way to provide nonlinear parameters for phenomenological models, compared with a lot of experiments.

## 4. Experimental Measurement for Nonlinear Signals 

As universal knows, nonlinear measurement of active devices is commonly realized. However, the measurement method is still not directly applicable to SAW duplexers, which is owing to:

(1) The nonlinear characteristics of SAW devices are quite weak. Thus, if there is no precaution and a high dynamic range of the testing system, the nonlinearity of SAW will be prone to be overwhelmed by the nonlinearity of measurement equipment and peripheral circuits.
(2) The power capacity of SAW devices is limited. Therefore, the imposed maximum input power of SAW devices is commonly no more than 36 dBm.
(3) Under the condition of large-signal measurement, it can be found that the frequency responses of SAW devices suffer from the self-heating effect. Therefore, it is critical to select a measurement method which is insensitive to self-heating effects.

Summarily, it is quite difficult to complete large-signal measurements for SAW duplexers. The weak nonlinearity level of SAW leads to measurement difficulty to observe the nonlinearity of SAW devices from the noise floor of the test system. Thus, to accomplish reliability and repeatability of nonlinear measurement, the impact of the testing system needs to be considered. However, the test equipment is usually complicated, and the calibration and verification of the test system are hard to be realized.

Even so, research teams including Qorvo, Murata, and Chiba University [3,6,8,9,10,11,12,13,22,31] have built and implemented the measurement systems on harmonics and intermodulation of nonlinearity for SAW devices. Moreover, our team also developed a harmonics and intermodulation measurement platform for SAW resonators, filters, and duplexers both on-chip and on PCB board.

### 4.1. Harmonics Measurement

Through the traditional equipment as a network analyzer, the S parameters of the device under test (DUT) can be obtained. However, the nonlinear harmonics measurement system under large continue wave (CW) signals can be conducted as follows.

**Figure 6:**
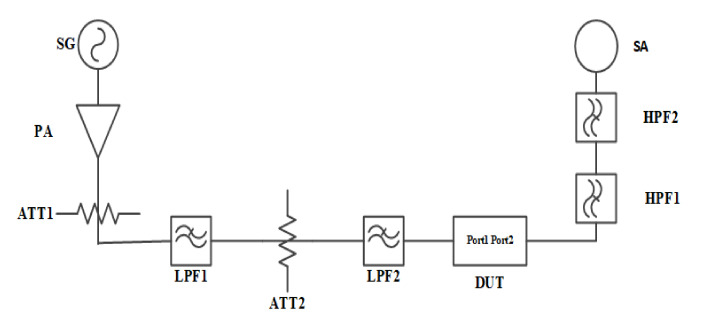
The nonlinear harmonic H2 and H3 measurement system.

The core methodology of the harmonic test is to feed a CW signal with a fundamental frequency to the DUT. Thereafter, the magnitude of signal with harmonic frequency from the output of the DUT to the coupling port can be tested and obtained. The harmonics measurement system is demonstrated in Figure 6. To achieve the test system with high dynamic range, accuracy, and reliability, the construction of the test system requires three key considerations. 

Firstly, it is the system distribution. Generally, the signal with fundamental frequency output by the PA is connected to the probe tip of the wafer probe station through a cable and fed to the resonator. Meanwhile, the spectrum analyzer with clock synchronization to signal generator is employed for harmonics characteristics of reflected spectrum measurement. Moreover, an attenuator ATT1 at the back of the PA is set to provide sufficient attenuation to protect the PA from harmful reflected energy. In addition, cascaded low-pass filters with attenuators are implemented behind the ATT1 to suppress the harmonic from PA for high system dynamic range. Besides, a coupler is placed near the test probe tip to conduct the reflected signal into the spectrum analyzer after the high-pass filter HPF1. Thus, tested nonlinear harmonic products can be derived by properly selecting the suitable attenuate value of the attenuator. 

Secondly, it is the frequency sweep setting. The center of the sweep frequency range of the signal generator (SG) is consistent with the frequency center of DUT. The bandwidth of the sweep range exceeds the coverage of the DUT passband. The spectrum analyzer is set to collect the reflected power of the resonator at the second harmonic H2, and third harmonic H3 spectrum.

Thirdly, it is the power setting. The system power outputs need to be calibrated. Thereafter, by appropriately selecting the output power of SG and gain of PA, the power reaching the DUT with 25 dBm, 20 dBm, 15 dBm respectively can be approached. Herein the power setting of 25 dBm is to simulate actual operating specifications. 

**Figure 7:**
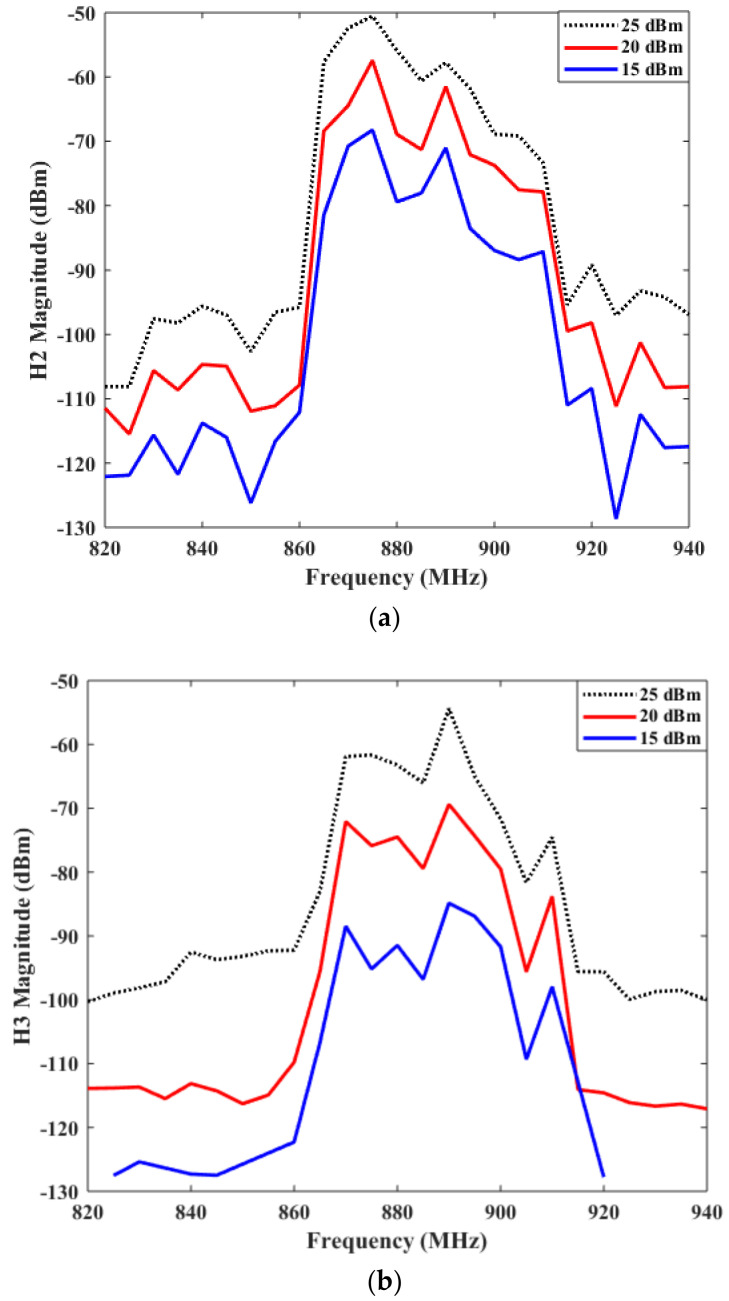
The measured harmonic nonlinear characteristics of Band 5 SAW filter. (**a**) The second harmonic response H2 (**b**) The third harmonic response H3.

We have employed the band 5 SAW filter as the DUT to accomplish the harmonic test system and approach the nonlinear effect test. The measurement results are depicted in Figure 7. As shown in Figure 7, we can observe that the measurement levels of harmonic effects H2 and H3 (at multiples of the fundamental frequency) are about −70 dBm and −90 dBm, respectively. And this situation is under the test condition that the SG1 source power is 15 dBm. In addition, we can also deduce from Figure 7 that the harmonic effect of the SAW filter is similar to the fundamental frequency characteristic curves of the SAW filter. That is, the harmonic response level is higher among the center harmonic frequency. Whereas, the frequency deviates from the center harmonic frequency, the harmonic response level weakens gradually.

### 4.2. Intermodulation Measurement

The second- and third- order intermodulation of SAW resonators are usually too weak to be observed. Indeed, the products are 70 to 120 dBc lower than the main signal power. Therefore, to observe and measure these weakly nonlinear signals, the dynamic range of the system must be higher than 120 dBc. This value even requires 160 dBc when an X-parameter vector network analyzer (X-VNA) is selected as the measurement terminal. Based on the previous nonlinear characteristics test experiments, we further conducted with the key issues to improve the dynamic range of the system as follows: 

Firstly, the appropriate frequency resolution should be determined. A smaller test resolution of frequency will effectively refine the dynamic range of the test system. Nevertheless, the peak value is hardly to be extracted when the frequency resolution is too low. Commonly, we set the frequency resolution 1 kHz of the measurement setup.

Secondly, a high level of out-of-band suppression of the filters should be implemented in the test system. The desired system dynamic range is hardly satisfied by utilizing a single LC filter. To attempt the ideal dynamic range, we independently designed, assembled and adopted suspended strip-line filters, cavity filters, and microstrip filters with a high level of stopband attenuation. 

Besides, many other factors also affect the dynamic range of the test system, such as the warm-up time of the system, whether the DUT is on the wafer or packaged, and the resolution of the spectrum analyzer, etc.

**Figure 8:**
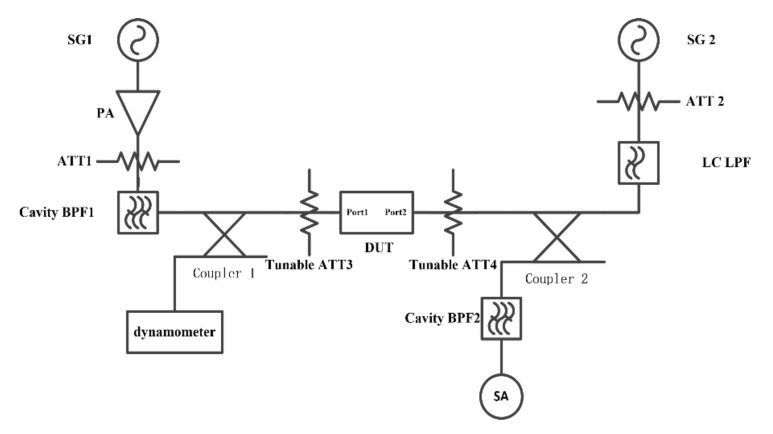
The nonlinear IMD2 (fTone1 + fTone2) measurement system.

**Figure 9:**
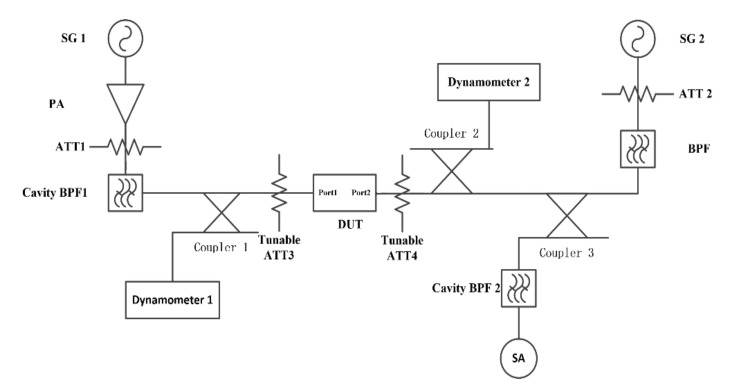
The nonlinear IMD3 (2fTone1 − fTone2) measurement system.

The IMD measurement system is conducted as depicted in Figure 8 and Figure 9 The two CW signals are input into Port I and Port II of DUT from two transmission paths respectively. The upper stopband simulated of Tone 1 and Tone 2 provoked from PA are attenuated by introducing the BPF 1 and BPF 2/3 (BPF 2 for IMD 2 measurement and BPF 3 for IMD 3 measurement). The attenuator ATT 1 and ATT 2 at the back of the PA are set to provide sufficient attenuation to protect the PA from harmful reflected energy. Moreover, a pair of attenuators is introduced at two ports of DUT. Nonlinear products with desired can be obtained by appropriate selection of the attenuate value of ATT 3 and ATT 4. A coupler is connected to the back of the DUT. Therefore, Tone 1 and Tone 2 are mixed at the DUT from two paths and IMD products generated. Then signals are output to the spectrum analyzer for detection through the coupling port of the coupler. Additionally, power meters are used to measure the power arriving at the SAW devices to derive the accurate power in the measurement.

### 4.3. Triple-Beat Measurement

In the investigation of high-order nonlinear effects, the nonlinear characteristics of triple beat should be further explored. The triple beat is in-band third-order nonlinear distortion, which is produced by three signals including the common two Tx signals and the other jammer signals.

**Figure 10:**
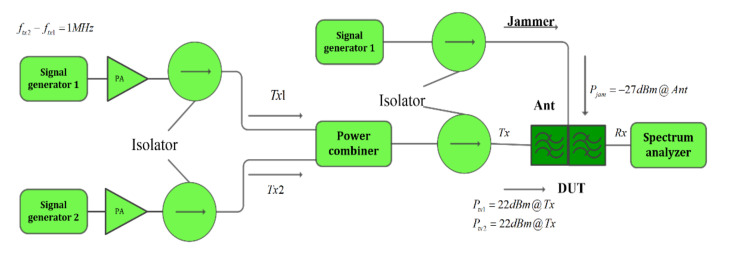
Setup for triple beat measurement.

This test focused on the transmission characteristics of the SAW filter in a more complex system of triple signal intermodulation transmission. For the in-band third-order nonlinear distortion demonstration, triple beat measurement, two-tone signals of the Tx band frequency are input to the Tx port in the duplexer. Among them, as shown in the Figure 10, two tones with SG1 and SG2 are combined through a microwave combiner first. Thereby, the combined signal entered the DUT duplexer from the Tx port. While the jammer signal tone3 (Jammer) of the Rx band frequency is simultaneously input to the antenna port. Thus, the triple beat signal can be measured by the spectrum analyzer at the Rx end.

## 5. Suppression for Nonlinear Effect

It is a difficult task to satisfy the high linearity requirements without compromising other performance and chip size. Existing studies are mainly carried out from the following aspects. 

On the one hand, from the basic structure of the resonator, this is one of the well-known countermeasures, is to replace one resonator with two series resonators, widely used in commercial equipment. For a given impedance, the replacement makes the area of the interdigital transducer four times larger, and thus the power density, which is directly related to the nonlinearity, can be decreased markedly. 

For example, for the PCS SAW diplexer in CDMA2000, a scheme of resonator splitting was proposed by Inoue et al. from Taiyo [27,28,29] for the first time to improve the triple beat effect in the passband of the SAW duplexer. The developed SAW duplexer achieved an extremely high linearity of approximately −90 dBc in the triple beat test, which is 20 dB better than the conventional duplexer, keeping the equivalent filter characteristics and the same packaging size.

In this method, by enlarging the area of the IDT, it can also improve heat dissipation. Thus, in the inspection of the heat dissipation, it can also weaken the nonlinear effect. Similarly, if the substrate of the SAW devices has better characters of heat dissipation, such as silicon and silicon carbide, etc., they may have a weaker nonlinear effect. This can be proved by the TCSAW and incredibly high-performance SAW (IHP SAW) devices with multilayer substrate [42,43]. 

On the other hand, based on the nonlinear generation mechanism, IDT materials are optimized [7,8,9,10,11,12,13,14]. As mentioned above, the third-order is closely related to the acoustic strain in the aluminum electrode, including the higher the crystallinity, the more obvious the nonlinear. When the electrode is laminated, the nonlinearity is suppressed. Moreover, the width of the electrode also has a great influence on the nonlinear effect, usually the wider the electrode width, the more obvious the nonlinear effect. 

## 6. Conclusions

SAW devices, as one of the indispensable components in the RF front-end of mobile phones, have a promising future in the area of communication. To satisfy the linear specification of the new communication technology, nonlinearity is one of the most important difficulties for SAW devices.

In the previous studies, the generation mechanism of nonlinearity in the substrate and electrode has been demonstrated. Accurate models including both many phenomenological models and precise models have been studied for nonlinear signals simulation. Furthermore, an experimental measurement setup has been developed both for harmonics and intermodulation nonlinear signals. Furthermore, suppression technology has been studied to some extent.

Even so, the study of the nonlinear effects of SAW devices is to be continued further, such as the faster and more precise simulation methods and more effective suppression technology for nonlinearity. Thanks to the recent research and all the further studies, it will push the development of future SAW devices and RF front-end modules. 

## Data Availability

Not applicable.
